# A universal molecular prognostic score for gastrointestinal tumors

**DOI:** 10.1038/s41525-021-00172-1

**Published:** 2021-02-04

**Authors:** Hideyuki Shimizu, Keiichi I. Nakayama

**Affiliations:** Department of Molecular and Cellular Biology, Medical Institute of Bioregulation, Kyushu University, Fukuoka, Japan

**Keywords:** Outcomes research, Colon cancer, Data processing

## Abstract

Colorectal and gastric cancers are a leading cause of cancer deaths in developed countries. Precise estimation of prognosis is important with regard to clinical decision making for individuals with such cancers. We here comprehensively compiled a complete atlas of prognostic genes based on an integrated meta-analysis of one of the largest assembled colorectal cancer cohorts. A simple yet robust machine learning approach was then applied to establish a universal molecular prognostic score (mPS_colon) that relies on the expression status of only 16 genes and which was validated with independent data sets. This score was found to be an independent prognostic indicator in multivariate models including cancer stage, to be valid independent of tumor characteristics or patient ethnicity, and to be also applicable to gastric cancer. We conclude that mPS_colon is a universal prognostic classifier for patients with gastrointestinal cancers and that it should prove informative for optimization of personalized therapy for such patients.

## Introduction

Cancer is the leading cause of death in developed countries, with an estimated 1.8 million new cases expected in the United States alone in 2020^1^. Among the many types of cancer, gastrointestinal cancers, including colorectal cancer (CRC) and gastric cancer, are among the most prevalent worldwide^2^.

Only a few biomarkers—including mutant *KRAS*, mutant *BRAF*, and microsatellite instability (MSI)—are currently recommended by expert panels for estimation of prognosis in CRC^3^, with the result that most patients receive similar treatment. The therapeutic strategy for CRC has thus been TNM staging, surgery, and chemotherapy. Current TNM criteria, however, may give rise to substantial under- or overtreatment of individuals with CRC. In addition, despite their receipt of similar treatment, CRC patients at the same stage show a wide range of outcomes. We hypothesized that such a difference in clinical outcome might be related to diverse transcriptome profiles of tumors. Identification of the molecular features of CRC that determine patient prognosis and stratification of patients on the basis of these features might be expected to inform the development of more effective clinical strategies and personalized therapies.

There is thus a growing need for new and efficient biomarkers to ensure optimal treatment of CRC patients. An ideal biomarker should be readily translated into clinical practice, identify patients who can be spared treatment or who can benefit from therapy, and, ultimately, support the implementation of precision medicine. Many analyses of CRC transcriptomes have been performed, and consensus molecular subtypes (CMSs) have recently been proposed by a multicenter initiative that undertook a comprehensive and cross-sectional comparison of such transcriptomes^4^. Almost all CRC tumors can thus be classified into one of four subtypes that show substantial biological differences: CMS1 is characterized by a high mutation rate, MSI, and pronounced activation of the immune system; CMS2 is epithelial in nature and manifests activation of WNT and MYC signaling pathways; CMS3 is also epithelial and shows overt metabolic dysregulation; and CMS4 exhibits marked transforming growth factor–β activity, stromal invasion, and angiogenesis^4^. However, CMS classification is not suited to prognostication, given that only CMS4 patients show a significant difference in overall survival (OS) and disease-free survival (DFS), with no prognostic difference being apparent among the other three subtypes^4^. These results highlight the need for a data-driven approach that does not rely on known biological findings and is focused on clinical outcomes.

Several previous studies have identified gene expression signatures that have a prognostic impact in individuals with stage 2 or 3 CRC. ColoGuide EX can stratify the prognosis of CRC patients on the basis of the expression levels of 13 genes^5^. However, this scoring system is specific to stage 2 patients and stratifies them into only two groups^5^. Oncotype DX Colon Recurrence Score is a 12 gene-based classifier that is applicable either to patients with stage 2 tumors that are mismatch repair (MMR) proficient or to those with stage 3 cancer^6,7^. The major drawback of this latter system is that it is protected by patents and only available with the use of an expensive test kit.

We recently developed the molecular Prognostic Score (mPS), a machine learning-based method for stratifying the prognosis of breast cancer patients^8^ on the basis of the expression levels of only 23 genes. Unlike existing prognostic classifiers for breast cancer (such as MammaPrint^9^ and Oncotype^10^), mPS is a universal indicator that can stratify prognosis regardless of breast cancer subtype or stage. We, therefore, aimed to develop a similarly simple but the accurate method to estimate the prognosis of CRC patients.

In this study, we exploited a large and multicenter series of gastrointestinal cancer samples to establish a robust molecular classification method on the basis of their transcriptomic profiles. We first identified prognostic genes differentially expressed between CRC and surrounding normal mucosa. We then trained a simple yet robust machine learning model and finally developed a 16-gene classifier, which we termed mPS for colon cancer (mPS_colon). We also demonstrate the applicability of mPS_colon to gastric cancer. mPS_colon is a universal prognostic indicator for gastrointestinal tumors across cancer types and patient ethnicities.

## Results

### Identification of differentially expressed and prognostic genes in CRC

We first identified genes that met both the following criteria: (1) genes that are differentially expressed between normal mucosa and CRC tissue (DEGs), and (2) genes that are associated with patient prognosis. We surveyed 4123 DEGs in the TCGA-COAD (CRC cohort of The Cancer Genome Atlas) data set (Supplementary Data 1). For the integrated identification of prognostic genes across different CRC cohorts, we collected eight public CRC data sets for which gene expression and prognostic data were available (Supplementary Data 2). We divided CRC patients of each cohort into two groups according to the median expression level for each identified DEG, and the hazard ratios (HRs) for relapse-free survival (RFS) were combined in a random-effects model (meta-analysis). The application of these two consecutive filters resulted in the identification of 77 prognostic and differentially expressed genes for CRC patients (Supplementary Data 3).

### Establishment of a 16 gene-based predictive score, mPS for CRC

**Fig. 1:**
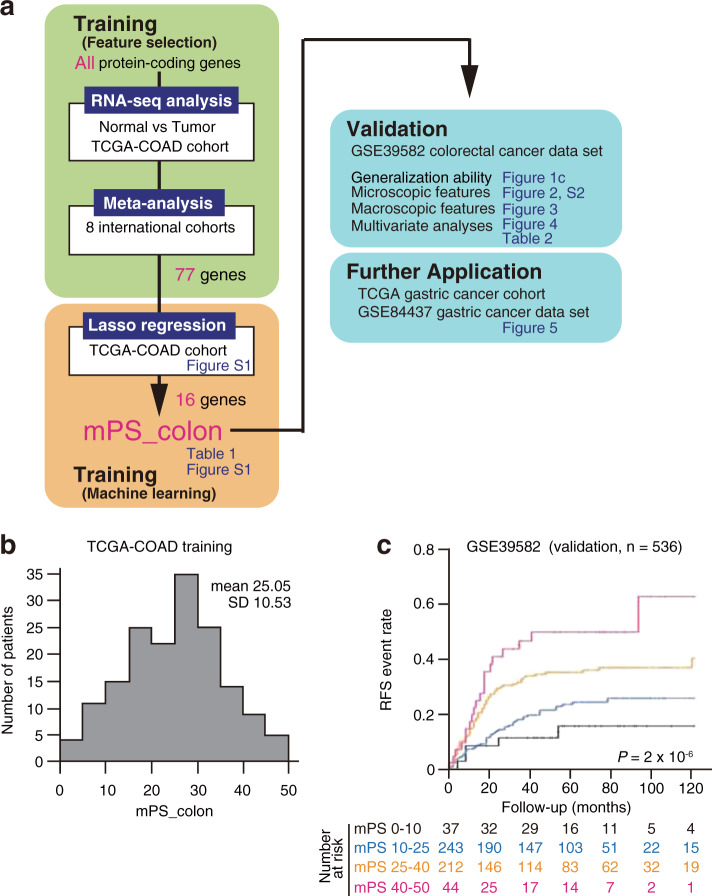
mPS_colon provides a simple and robust estimation of prognosis for colorectal cancer (CRC) patients. **a** All genes were tested for their potential as prognostic genes with the use of nine independent multicenter data sets. Genes were filtered to identify differentially expressed genes between normal mucosa and CRC tissue with the use of the TCGA-COAD data set, and the identified genes were subjected to a meta-analysis with eight additional cohorts. A machine learning approach (lasso regression) then extracted 16 genes that are important for the prediction of relapse. The robustness of mPS_colon was demonstrated with various CRC subsets. Finally, we found that mPS_colon also estimates the prognosis of individuals with gastric cancer. **b** mPS_colon distribution in the TCGA-COAD training cohort. **c** Kaplan–Meier curves of relapse-free survival (RFS) for the GSE39582 CRC test cohort based on mPS_colon. The log-rank *P* value is shown.

We next attempted to stratify the prognosis of CRC patients according to the expression levels of these 77 genes. We applied a simple yet robust machine learning method known as lasso regression to TCGA-COAD training data in order to predict 5-year DFS (Fig. 1a). This approach resulted in the extraction of 16 of the 77 genes that are important for estimation of prognosis and provided weight for each (Table 1). We named this weighted summation mPS_colon, and it was designed to range from 0 to 50, with an average of 25.05 (Fig. 1b). A representative example of mPS_colon calculation is shown in Supplementary Fig. 1a.

**Table 1:** The 16 genes necessary and sufficient for calculation of mPS_colon.

Entrez Gene ID	Gene name	Official name	Weight	High	Low
8863	PER3	Period circadian regulator 3	5.100	1	0
339105	PRSS53	Serine protease 53	4.569	1	0
3801	KIFC3	Kinesin family member C3	3.328	1	0
7306	TYRP1	Tyrosinase related protein 1	2.078	1	0
118663	BTBD16	BTB domain containing 16	1.337	1	0
1136	CHRNA3	Cholinergic receptor nicotinic alpha 3 subunit	1.047	1	0
55366	LGR4	Leucine-rich repeat-containing G protein-coupled receptor 4	4.597	0	1
55646	LYAR	Ly1 antibody reactive	4.439	0	1
10576	CCT2	Chaperonin containing TCP1 subunit 2	4.404	0	1
898	CCNE1	Cyclin E1	4.226	0	1
3276	PRMT1	Protein arginine methyltransferase 1	4.155	0	1
10420	TESK2	Testis associated actin remodeling kinase 2	4.060	0	1
130574	LYPD6	LY6/PLAUR domain containing 6	3.960	0	1
2150	F2RL1	F2R like trypsin receptor 1	1.465	0	1
55165	CEP55	Centrosomal protein 55	1.061	0	1
54414	SIAE	Sialic acid acetylesterase	0.174	0	1

For the first six genes, patients with a high level of expression (above the median) are assigned a score of 1. Conversely, for other genes, patients with a low level of expression (below the median) are assigned a score of 1. A representative calculation is shown in Supplementary Fig. 1a.

On the basis of this scoring method, we stratified CRC patients in the training data set into four groups (<10, 10–25, 25–40, and >40), and we found that the higher the score, the more likely the patients were to experience relapse (Supplementary Fig. 1b). To test the robustness of mPS_colon, we adopted another independent cohort, GSE39582^11^, which is the largest publicly available CRC data set, for external validation. We found that mPS_colon also stratified RFS in this validation cohort (Fig. 1c), and we, therefore, concluded that mPS_colon is a robust prognostic indicator for CRC patients.

### mPS_colon is a universal prognostic scoring system

**Fig. 2:**
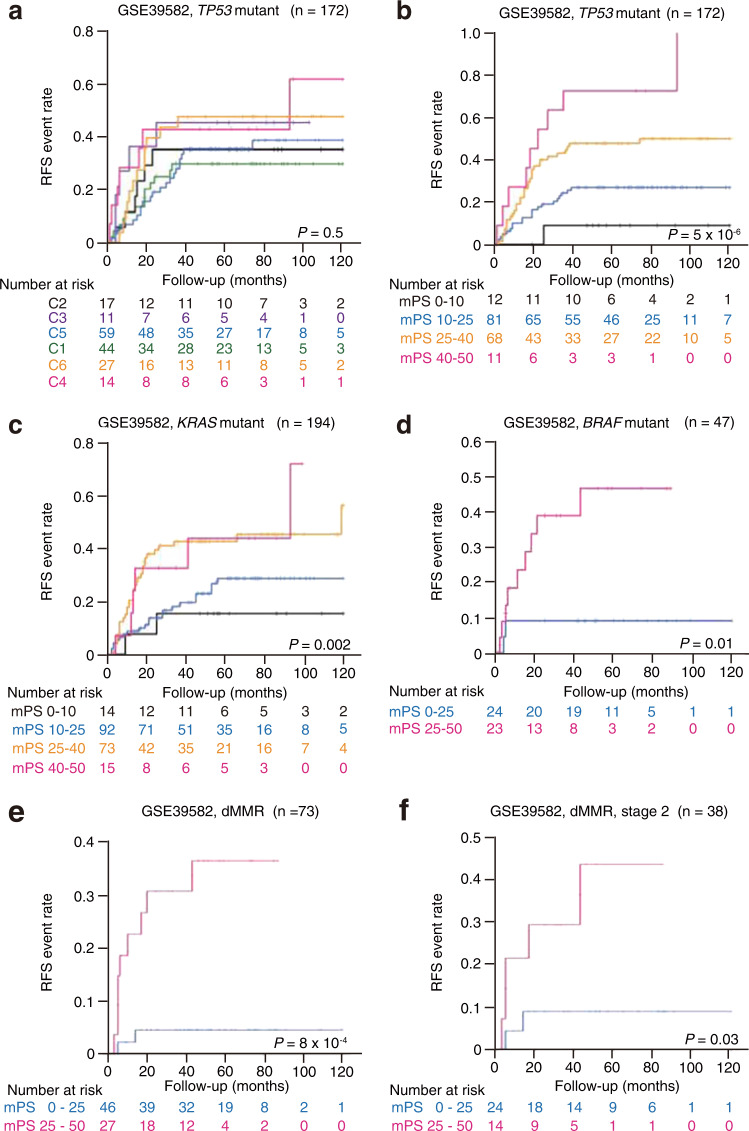
mPS_colon stratifies colorectal cancer patients in the GSE39582 cohort regardless of molecular status. **a** Kaplan–Meier curve of relapse-free survival (RFS) for *TP53* mutation-positive patients in the GSE39582 cohort on the basis of molecular subtypes defined previously^11^. The log-rank *P* value is shown. **b**–**f** Kaplan–Meier curves of RFS according to mPS_colon for patients harboring *TP53* (**b**), *KRAS* (**c**), or *BRAF* (**d**) mutations as well as for those with mismatch repair-deficient (dMMR) tumors (**e**) or those with dMMR tumors at stage 2 (**f**). The log-rank *P* values are shown. Only patients with available information are included.

CRC has been well investigated as a model of multistage carcinogenesis^12^. In particular, about half of CRC tumors have been found to harbor *TP53* mutations, with the frequency of such mutations being higher in cancers of the distal colon and rectum than in those of the proximal colon^13^. In accordance with this observation, 53.75% of CRC patients in the TCGA cohort harbor *TP53* mutations. Any prognostic indicator would therefore need to be applicable to *TP53* mutation-positive patients. The providers of the GSE39582 data set have proposed molecular subtypes (C1–C6) of CRC based on unsupervised consensus hierarchical clustering^11^. However, this classification system is not able to stratify patients with *TP53* mutations with regard to RFS (Fig. 2a). In contrast, mPS_colon was able to stratify CRC patients harboring these mutations (Fig. 2b).

Mutations in *KRAS* and *BRAF* are also frequently present in CRC patients^14^. We surveyed patients in the GSE39582 cohort for the seven most frequent mutations in codons 12 or 13 of *KRAS*^15^ and for the c.1799T > A (p.V600E) mutation of *BRAF*, and we found that mPS_colon was also able to stratify the prognosis of CRC patients with these mutations (Fig. 2c, d).

Among molecular markers developed previously for CRC characterization and prognosis estimation, MSI dependent largely on deficient MMR is the only one reproducibly found to be a significant prognostic factor in early CRC by both a meta-analysis and a prospective trial^3,16^. We found that mPS_colon could stratify the prognosis of CRC patients with either MMR-deficient (dMMR) (Fig. 2e) or MMR-proficient (pMMR) (Supplementary Fig. 2a) tumors. Of note, whereas Oncotype DX is not applicable to stage 2 patients with dMMR tumors, mPS_colon was able to stratify these patients (Fig. 2f), suggestive of broader applicability of mPS_colon.

Other molecular features of CRC include its CpG island methylator phenotype (CIMP) and chromosome instability (CIN). CIMP is an indicator based on DNA methylation status, whereas CIN is based on chromosomal aberrations^17^. The prognosis of subsets of CRC patients classified according to CIMP (Supplementary Fig. 2b, c) or CIN (Supplementary Fig. 2d, e) could be further stratified by mPS_colon.

**Fig. 3:**
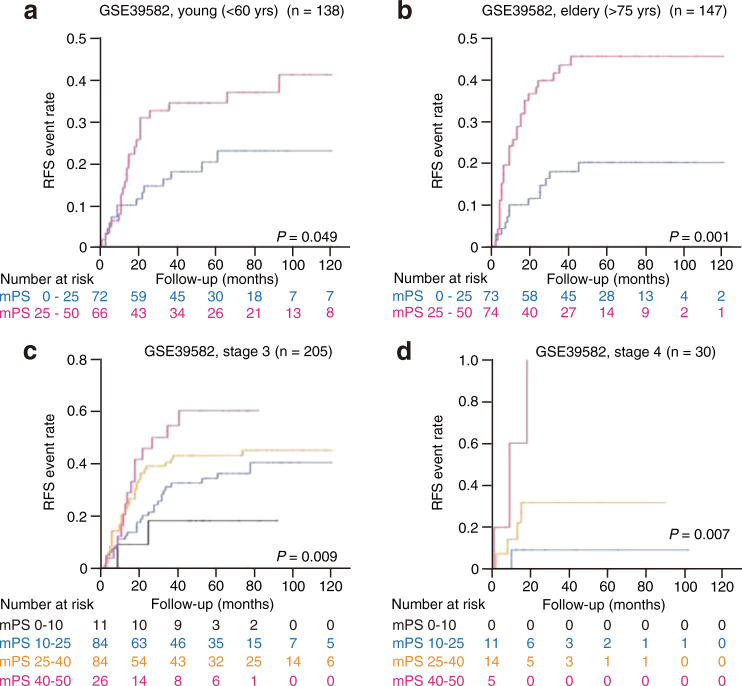
mPS_colon stratifies colorectal cancer patients regardless of age or disease stage. **a**, **b** Kaplan–Meier curves of relapse-free survival (RFS) for patients aged <60 years (**a**) or >75 years (**b**) in the GSE39582 cohort according to mPS_colon. The log-rank *P* values are shown. **c**, **d** Kaplan–Meier curves of RFS for patients with stage 3 (**c**) or stage 4 (**d**) disease in the GSE39582 cohort according to mPS_colon. The log-rank *P* values are shown. Only patients with available information are included.

We found that mPS_colon could also stratify prognosis in both young (<60 years old) (Fig. 3a) and elderly (>75 years) (Fig. 3b) CRC patients. In addition, the prognosis for patients of TNM stage 3 (Fig. 3c) or stage 4 (Fig. 3d) was stratified by mPS_colon. These various findings indicate that mPS_colon is a universally applicable prognostic indicator without regard to molecular subtypes and macroscopic clinical features of CRC.

**Fig. 4:**
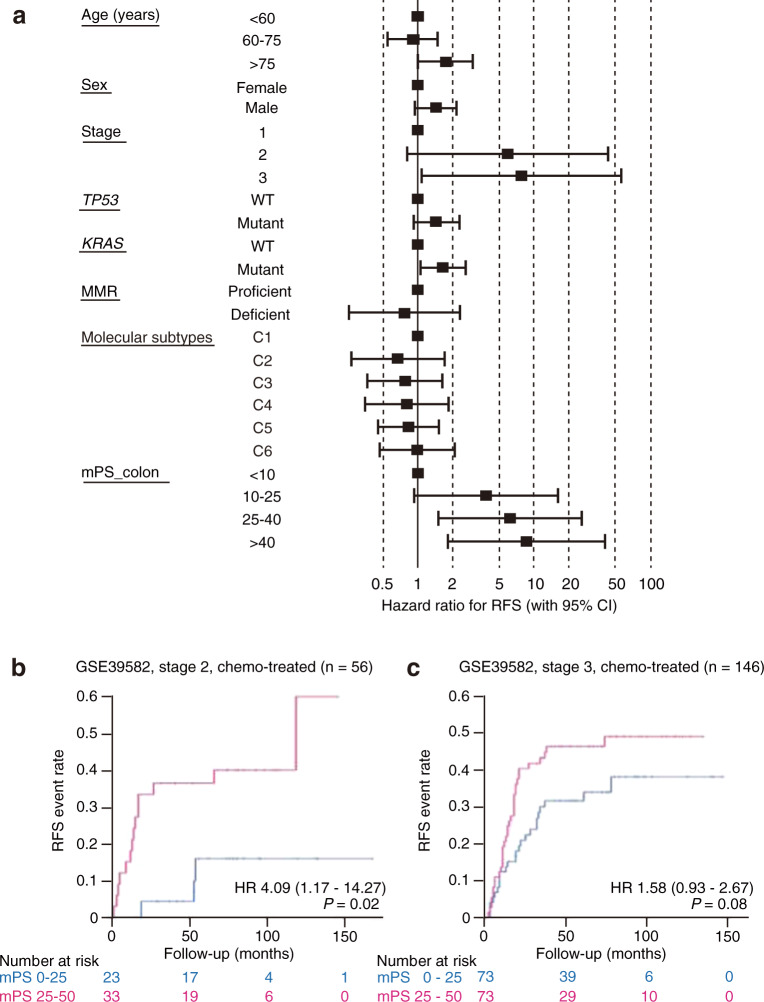
mPS_colon is an independent prognostic indicator. **a** Multivariate analysis of relapse-free survival (RFS) for metastasis-free colorectal cancer patients in the GSE39582 cohort. Hazard ratios (HRs) and their 95% confidence intervals (CIs) were calculated with the Cox proportional hazard model. **b**, **c** Kaplan–Meier curves of RFS according to mPS_colon for stage 2 (**b**) or stage 3 (**c**) patients treated with chemotherapy. HRs (with 95% CIs) and log-rank *P* values are shown. Only patients with available information are included.

To assess whether mPS_colon is a prognostic indicator for CRC patients independent of various clinicopathologic features, we performed the multivariate analysis with the use of a Cox proportional hazard model including patient age and sex, disease stage, *TP53* and *KRAS* mutation status, MMR status, and molecular subtypes proposed by the providers of the GSE39582 data set (Fig. 4a, Table 2). We found that mPS_colon was able to stratify the recurrence risk of CRC patients independently of these features, with its impact on RFS being similar to that of TNM stage classification. Of note, mPS_colon was the most powerful prognostic factor among the known microscopic indicators (*TP53* mutation, *KRAS* mutation, MMR status, and molecular subtypes).

**Table 2:** Univariate and multivariate analyses for relapse-free survival of metastasis-free patients with colorectal cancer in the GSE39582 cohort.

	Univariate			Multivariate		
	HR	95% CI	*P*	HR	95% CI	*P*
*Age (years)*						
<60	1 (ref)			1 (ref)		
60–75	0.901	0.601–1.35	0.614	0.933	0.561–1.55	0.789
>75	1.14	0.732–1.78	0.558	1.71	0.989–2.95	0.0548
*Sex*						
Female	1 (ref)					
Male	1.39	0.988–1.95	0.0584	1.44	0.949–2.20	0.0865
*Stage*						
1	1 (ref)			1 (ref)		
2	9.387	1.30–67.7	0.0263*	6.00	0.821–43.8	0.0775
3	17.5	2.43–126	0.00446**	7.83	1.07–57.1	0.0424*
*TP53*						
Wild type	1 (ref)			1 (ref)		
Mutant	1.25	0.845–1.86	0.261	1.39	0.890–2.17	0.148
*KRAS*						
Wild type	1 (ref)			1 (ref)		
Mutant	1.54	1.10–2.17	0.0129*	1.72	1.09–2.70	0.0196*
MMR						
Proficient	1 (ref)			1 (ref)		
Deficient	0.500	0.267–0.926	0.0275*	0.776	0.255–2.36	0.655
*Molecular subtype*						
C1	1 (ref)			1 (ref)		
C2	0.558	0.308–1.01	0.0546	0.674	0.270–1.68	0.398
C3	0.791	0.426–1.47	0.458	0.754	0.357–1.59	0.459
C4	1.80	1.02–3.17	0.0423*	0.805	0.353–1.84	0.605
C5	0.867	0.534–1.41	0.564	0.848	0.465–1.55	0.590
C6	1.42	0.819–2.46	0.212	1.06	0.513–2.20	0.872
*mPS_colon*						
<10	1 (ref)			1 (ref)		
10–25	1.74	0.693–4.36	0.239	3.87	0.926–16.2	0.0637
25–40	2.95	1.19–7.32	0.0194*	6.18	1.49–25.7	0.0123*
>40	4.30	1.60–11.6	0.00391**	8.46	1.79–40.0	0.00704**

The hazard ratio (HR), its 95% confidence interval (CI), and *P* value are shown. *P < 0.05, **P < 0.01.

Stage 2 and 3 patients often receive adjuvant chemotherapy. We found that CRC patients with a high mPS_colon score at either of these stages have a higher recurrence rate after chemotherapy compared with those with a low score (Fig. 4b, c). These results suggest that mPS_colon also has the potential to identify high-risk patients among those receiving adjuvant chemotherapy and that such patients with a high mPS_colon score may need more frequent follow-up examinations or additional therapy, or both.

### mPS_colon stratifies patients with gastric cancer

Stomach cancer is responsible for an estimated 783,000 deaths annually worldwide, making it the third leading cause of cancer deaths^18^. The stomach and colon share a similar embryological origin and similar cancer histopathology. Although gastric cancer is the second most common gastrointestinal cancer after CRC, no molecular score for stratification of the prognosis of gastric cancer patients has been adopted clinically.

**Fig. 5:**
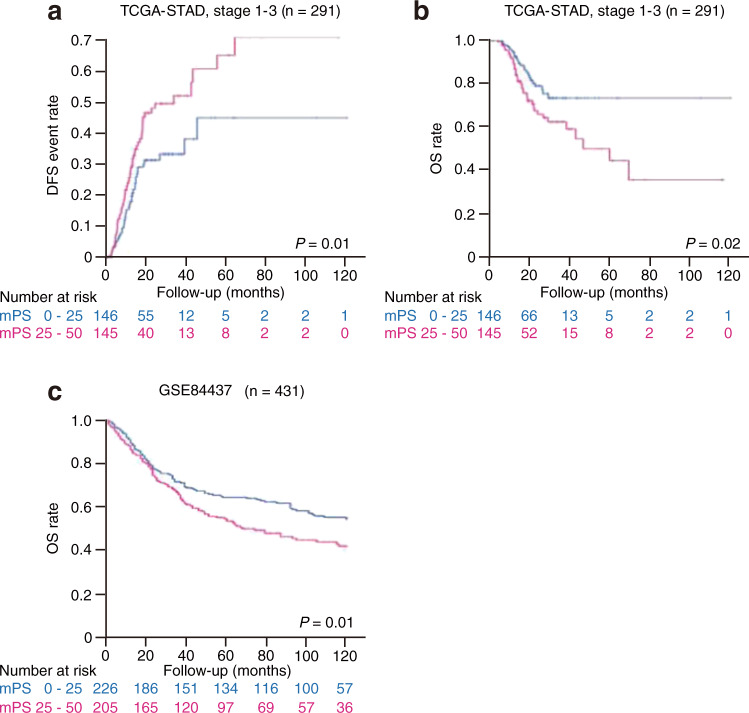
mPS_colon universally stratifies patients with gastrointestinal tumors. **a**, **b** Kaplan–Meier curves of disease-free survival (DFS) (**a**) or overall survival (OS) (**b**) according to mPS_colon for metastasis-free gastric cancer patients in the TCGA-STAD cohort. The log-rank *P* values are shown. **c** Kaplan–Meier curves of OS according to mPS_colon for East Asian gastric cancer patients in the GSE84437 cohort. Only patients with available information are included.

We hypothesized that mPS_colon, which was trained with data for the CRC cohort (mostly Caucasian) of TCGA, might also be applicable to gastric cancer, given the common characteristics shared by gastrointestinal cancers. Application of mPS_colon to patients in the gastric cancer cohort of TCGA (TCGA-STAD) without metastasis revealed that both DFS (Fig. 5a) and OS (Fig. 5b) were significantly stratified. These results thus indicate that mPS_colon also partially stratifies gastric cancer patients.

Given that the incidence of gastric cancer shows regional differences, being highest in East Asia^18^, we also assessed the utility of mPS_colon with the GSE84437 cohort, which is the largest gastric cancer data set currently available to the public and was derived from patients in South Korea. We found that mPS_colon also stratified the prognosis of these East Asian patients (Fig. 5c). Our results thus suggested that mPS_colon is a simple yet robust classifier for gastrointestinal tumors across cancer types and patient ethnicities.

## Discussion

In this study, we made use of public data to comprehensively identify prognosis-related genes with a meta-analysis of >1200 patients and we then developed a universal prognostic classifier for gastrointestinal cancers, mPS_colon, with the use of machine learning technology. Although it is simple to apply, mPS_colon is a robust prognostic indicator for gastrointestinal tumors across cancer types and patient ethnicities.

This score is calculated from the binary expression status of only 16 genes. It is of note that well-characterized genes associated with CRC, including *APC* and *KRAS*, are not included among the prognostic genes we identified, suggestive of strong experimenter bias in previous studies that focused mostly on mutation status. In contrast, we adopted a strategy to identify in systematic manner genes that are most associated with patient prognosis at the transcriptome level with an unbiased machine learning approach. Given that mPS_colon was developed in a manner independent of the mechanistic contribution of each gene, most of the 16 genes identified in our study are not well characterized with regard to how they might affect the prognosis of patients with gastrointestinal cancers.

The biological relevance of these novel genes remains to be determined, but a few studies have suggested possible relations between some of these genes and disease. The nicotinic cholinergic receptor gene *CHRNA3* is highly expressed in the human colon and small intestine^19^; the product of *CCT2* was shown to cooperate with Gli1 and Hedgehog in the development of CRC^20^; CEP55 contributes to a feedback loop with the master transcription factor FOXM1 in malignant transformation^21^; and the prognosis of patients with a high level of *TYRP1* expression in their tumors was found to be poor in a small CRC cohort^22^, consistent with our present findings. No previous biological analysis has examined a possible relationship between CRC and seven of the 16 genes (*PRSS53*, *KIFC3*, *BTBD16*, *TESK2*, *LYPD6*, *F2RL1*, and *SIAE*). Further studies are thus warranted to elucidate the biological relevance of each of the 16 genes in the context of CRC.

The 16 prognostic genes found here to be related to colorectal and gastric cancer show no overlap with the 23 prognostic genes that we previously identified for breast cancer by a similar data-driven approach^8^. The transcriptomic characteristics of cancer would not be expected to be highly tissue-specific, given the common mechanisms thought to contribute to the development of various cancer types. Again, it should be emphasized, however, that most of the prognostic genes we identified in both the present and our previous^8^ study have not been extensively investigated in the field of cancer research. Further advances in unbiased methodology, together with the increasing accumulation of data, should lead to a more precise stratification of cancer patients for personalized medicine.

Although mPS_colon is a simple 16 gene-based classifier, it is substantially superior to the molecular subtypes proposed by the providers of the GSE39582 data set, especially for patients harboring *TP53* mutations (Fig. 2a, b). The simplicity of mPS_colon would minimize the effort required for and cost of its application to clinical practice. We have also developed a Web tool to help clinicians who are unfamiliar with computational skills to perform the necessary calculations (https://hideyukishimizu.github.io/mPS_GI). The model we present is highly interpretable, making it easy to identify transcriptome patterns that may increase the risk for disease recurrence. The development of drugs that target the identified prognostic genes may reduce such risk in the future.

Other transcriptome-based methods, such as ColoGuide EX, have been developed for the prediction of the prognosis of patients with CRC. However, whereas these previous methods categorize patients into binary classes, mPS_colon is able to stratify patients into several groups. Furthermore, whereas many of the existing methods can be applied only to patients at specific stages of the disease or require certain platforms (as is the case for Oncotype DX Colon), our scoring system is applicable to a broader range of patients and is platform-independent, with both RNA-sequencing data (TCGA-COAD and TCGA-STAD) and microarray data (GSE39582 and GSE84437) being acceptable.

There are three main limitations of our study. First, all analyses were performed in a retrospective manner. Demonstration of the effectiveness of our prognostic stratification method for clinical use will require prospective evaluation of patients with gastrointestinal cancers and their prognosis for 10 years. Second, some information may be lost in the binarization process, which we used to maximize available data and to develop a platform-independent score. The availability of more RNA-sequencing data associated with clinical outcomes in the future may allow the application of other processes (such as meta-regression) that produce a better prognosis classifier. Third, although mPS_colon can stratify patient outcomes, it remains difficult to determine the best treatment for each group of patients. This problem is not limited to mPS_colon, however. It applies to all prognostic methods in the developmental stage, with the determination of an optimal treatment for each patient group generally requiring many years of further study. In breast cancer, for example, attempts to stratify patient prognosis on the basis of molecular markers have a relatively long history. A representative method, MammaPrint, was proposed in 2002^23^, but interventions for the poor and good prognosis groups were not proposed until 2016, after the performance of several clinical trials^24^. Our mPS_colon will also undergo multiple validation and intervention trials in the future in order to determine the appropriate course of treatment for each category of patients.

In summary, we have developed a universal prognostic indicator for gastrointestinal cancers that is based on the expression status of only 16 genes. The resulting score, mPS_colon, is able to stratify patients with gastrointestinal cancers, and further detailed characterization of each group of such patients categorized by mPS_colon may provide clues for future personalized medicine. In addition, many of the 16 prognostic genes identified have not been characterized in the context of colorectal or gastric cancer, and further studies of these genes may therefore provide insight into the development and progression of gastrointestinal cancers.

## Methods

### Study design and data sources

An overview of the development of the mPS_colon system is shown in Fig. 1a. We performed an integrated retrospective analysis of nine independent CRC cohorts for the establishment of mPS_colon. The initial analysis (Supplementary Data 1) was conducted with the TCGA-COAD data set (*n* = 382)^25^. We then performed a meta-analysis (random-effects model) to determine prognosis-related genes in a large combined multicenter cohort consisting of eight international CRC data sets (GSE14333^26^, *n* = 290; GSE17536^27^, *n* = 177; GSE29623^28^, *n* = 65; GSE33113^29^, *n* = 90; GSE37892^30^, *n* = 130; GSE38832^31^, *n* = 122; GSE72970^32^, *n* = 124; and GSE87211^33^, *n* = 203) that include 1201 patients (Supplementary Data 2). These cohorts were selected because of their inclusion of a substantial number of CRC patients (>50 each) with both clinicopathologic and prognostic data available. We then adopted half of the TCGA-COAD cohort as a training set (TCGA-COAD-training) and the other half as an internal validation set (TCGA-COAD-validation) for machine learning to develop mPS_colon (Fig. 1a). For external validation, another independent data set, GSE39582^11^ (*n* = 585), which is the largest public CRC data set available, was used. We extracted data with an RFS > 0 (*n* = 536). The clinicopathologic characteristics of each cohort are described in the original reports^11,25–33^. Molecular subtypes for the GSE39582 cohort were calculated by the providers and included in the public data set^11^. For the application of mPS_colon to gastric cancer, we adopted the stomach cancer data set of TCGA (TCGA-STAD, *n* = 415) together with the largest gastric cancer data set publicly available (GSE84437, *n* = 433^34^, which was established by researchers at Yonsei University in South Korea. We extracted data with a DFS > 0 (TCGA-STAD, *n* = 291; GSE84437, *n* = 431). Both the GSE39582 (CRC) and TCGA-STAD (stomach cancer) cohorts have a sufficient number of patients to achieve a statistical power of at least 80%.

### Predictive modeling

We first identified DEGs between normal mucosa and CRC samples deposited in TCGA-COAD (Supplementary Data 1). We performed this analysis with the TCGAbiolinks R package as recommended by the developer. In brief, we fitted a negative binomial generalized log-linear model to the read counts for each gene (TCGAanalyze_DEA function with method = ‘glmLRT’ option). We defined DEGs as genes with a false discovery rate (FDR) of <0.01 and absolute log_2_[fold change] of >1, meaning that the expression level differs by a factor of >2 or <0.5 between normal and cancer samples. We did not use the data in the other columns of Supplementary Data 1, including log[CPM], to define DEGs.

We next downloaded eight public CRC data sets from GEO (Supplementary Data 2) and examined the relation between DEGs and prognosis by meta-analysis (random-effects model). For this analysis, we used the median value as the cutoff between low and high expression levels for each DEG in each cohort. A total of 77 genes was identified after the application of these two consecutive filters (Supplementary Data 3).

We then used the TCGA-COAD data set for the establishment of the prognostic classifier. The expression status (X) of the 77 genes was first transformed to “Gene_Score” on the basis of the expression level and integrated HR for each gene with the following step function (Eq. (1))

1 $${\rm{Gene}}\_{\rm{Score}} = \left\{ {\begin{array}{*{20}{c}} {1,\,{\rm{if}}\,X\,{\rm{is}}\,{\rm{LOW}}\,{\rm{and}}\,{\rm{integrated}}\,{\rm{HR}} < 1} \\ {1,\,{\rm{if}}\,X\,{\rm{is}}\,{\rm{HIGH}}\,{\rm{and}}\,{\rm{integrated}}\,{\rm{HR}} > 1} \\ {0,\,{\rm{if}}\,X\,{\rm{is}}\,{\rm{LOW}}\,{\rm{and}}\,{\rm{integrated}}\,{\rm{HR}} > 1} \\ {0,\,{\rm{if}}\,X\,{\rm{is}}\,{\rm{HIGH}}\,{\rm{and}}\,{\rm{integrated}}\,{\rm{HR}} < 1} \end{array}} \right.$$

We truncated the clinical information to 5 years (60 months), built a simple learning algorithm, L1 (lasso) regression, and trained the model to predict the defined period, 60 minus DFS in months. Lasso regression tends to be unstable when the sample size is limited compared with the number of variables. We, therefore, narrowed down the promising genes by the application of two consecutive filters before lasso regression. A similar approach (DEGs + meta-analysis followed by lasso) was also adopted in a recent study^35^.

We generated the machine learning model with the use of the Python-based Keras library. It extracted 16 genes necessary to predict the prognosis of CRC patients. We defined Gene_Weight of these 16 genes in order to maximize the value to 50. Importantly, in this initial training, we used data only from CRC patients. However, we found that the 16 gene-based molecular score is also applicable to stomach cancer patients.

### Statistics

Kaplan–Meier plots were generated with the survival R package. In general, we used a four-way split for the survival curves. When the number of patients in each category was small, we used a two-way split, with the exception of the data in Fig. 3d (which were split four ways for easy comparison with Fig. 3c). We used the meta R package for meta-analysis (random-effects model). The l1 lambda parameter of the lasso regression was set to 0.5 because with this value among the tested values (0.01, 0.05, 0.1, 0.5, and 1) the model yielded the best prediction with the TCGA-COAD-validation cohort. For the external validation of mPS_colon, we truncated the survival data at 10 years. We computed time from the date of diagnosis to the date of the event. Survival outcomes were compared with the log-rank test. The HR and its 95% confidence interval (CI) were calculated by univariate or multivariate Cox regression. Statistical significance was determined at a two-sided *P* value of <0.05, with the exception of the initial RNA-sequencing data analysis (FDR of <0.01, with the use of the TCGAbiolinks R package^36^) and subsequent meta-analysis (*P* value of <0.01).

### Ethics

Ethical approval was not needed because the datasets are publicly available.

### Reporting summary

Further information on research design is available in the Nature Research Reporting Summary linked to this article.

## Supplementary information

Supplementary Information

Supplementary Data 1

Supplementary Data 2

Supplementary Data 3

Reporting Summary

## Data Availability

All the data analyzed in this study were downloaded from cBioPortal or GEO. The links for each data are provided in Supplementary Data 2.

## Supplementary information

**Supplementary information** The online version contains supplementary material available at 10.1038/s41525-021-00172-1.

## Code availability

Algorithms used for the creation of mPS_colon are detailed in the “Methods” section. A Web-based tool we generated in this study is freely available on our GitHub page.
