# Supplementary material for: A universal molecular prognostic score for gastrointestinal tumors
Source: NPJ Genom Med. 2021 Feb 4;6:6. doi: 10.1038/s41525-021-00172-1 (PMC7862603; doi:10.1038/s41525-021-00172-1)
Supplement: Supplementary file 1 — Supplementary Information [file 41525_2021_172_MOESM1_ESM.pdf]

SUPPLEMENTARY INFORMATION

A universal molecular prognostic score for gastrointestinal tumors

Hideyuki Shimizu, Keiichi I. Nakayama

Supplementary Data 1–3

Supplementary Figures 1 and 2

SUPPLEMENTARY DATA

Supplementary Data 1 Differentially expressed genes (DEGs) between cancer cells and surrounding mucosa in TCGA-COAD patients. (Provided as an Excel file and not included here.)

Supplementary Data 2 International colorectal cancer (CRC) cohorts used in this research. (Provided as an Excel file and not included here.)

Supplementary Data 3 Prognosis-related differentially expressed genes in colorectal cancer patients. (Provided as an Excel file and not included here.)

SUPPLEMENTARY FIGURES

a TCGA-NH-A8F7

Gene name	Expression	Gene_Score	Gene_Weight	Score x Weight
PER3	Above median	1	5.100	5.100
PRSS53	Below median	0	4.569	0
KIFC3	Below median	0	3.328	0
TYRP1	Above median	1	2.078	2.078
BTBD16	Below median	0	1.337	0
CHRNA3	Above median	1	1.047	1.047
LGR4	Below median	1	4.597	4.597
LYAR	Below median	1	4.439	4.439
CCT2	Below median	1	4.404	4.404
CCNE1	Above median	0	4.226	0
PRMT1	Below median	1	4.155	4.155
TESK2	Below median	1	4.060	4.060
LYPD6	Above median	0	3.960	0
F2RL1	Above median	0	1.465	0
CEP55	Below median	1	1.061	1.061
SIAE	Below median	1	0.174	0.174

mPS 31.115

b TCGA-COAD training

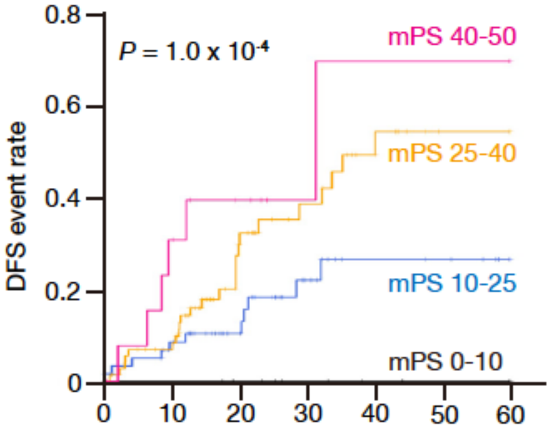

Number at risk							
mPS 0-10	15	14	12	9	7	6	6
mPS 10-25	62	51	35	19	12	11	7
mPS 25-40	74	64	28	18	9	4	4
mPS 40-50	14	9	5	2	1	1	1

Shimizu et al. Supplementary Figure S1

Supplementary Fig. 1 Calculation and stratification of mPS_colon in the TCGA-COAD training cohort. a Representative calculation of mPS_colon (for patient TCGA-NH-A8F7). For the first six genes, Gene_Score is 1 if the gene is expressed at a level higher than the median in the cancer; for the remaining 10 genes, Gene_Score is 1 if the gene is expressed at a level lower than the median. Otherwise, Gene_Score is set to 0. mPS_colon is calculated by summation of the weighted scores. **b** Kaplan-Meier curves of disease-free survival (DFS) for the TCGA-COAD training cohort based on mPS_colon. The log-rank *P* value is shown.

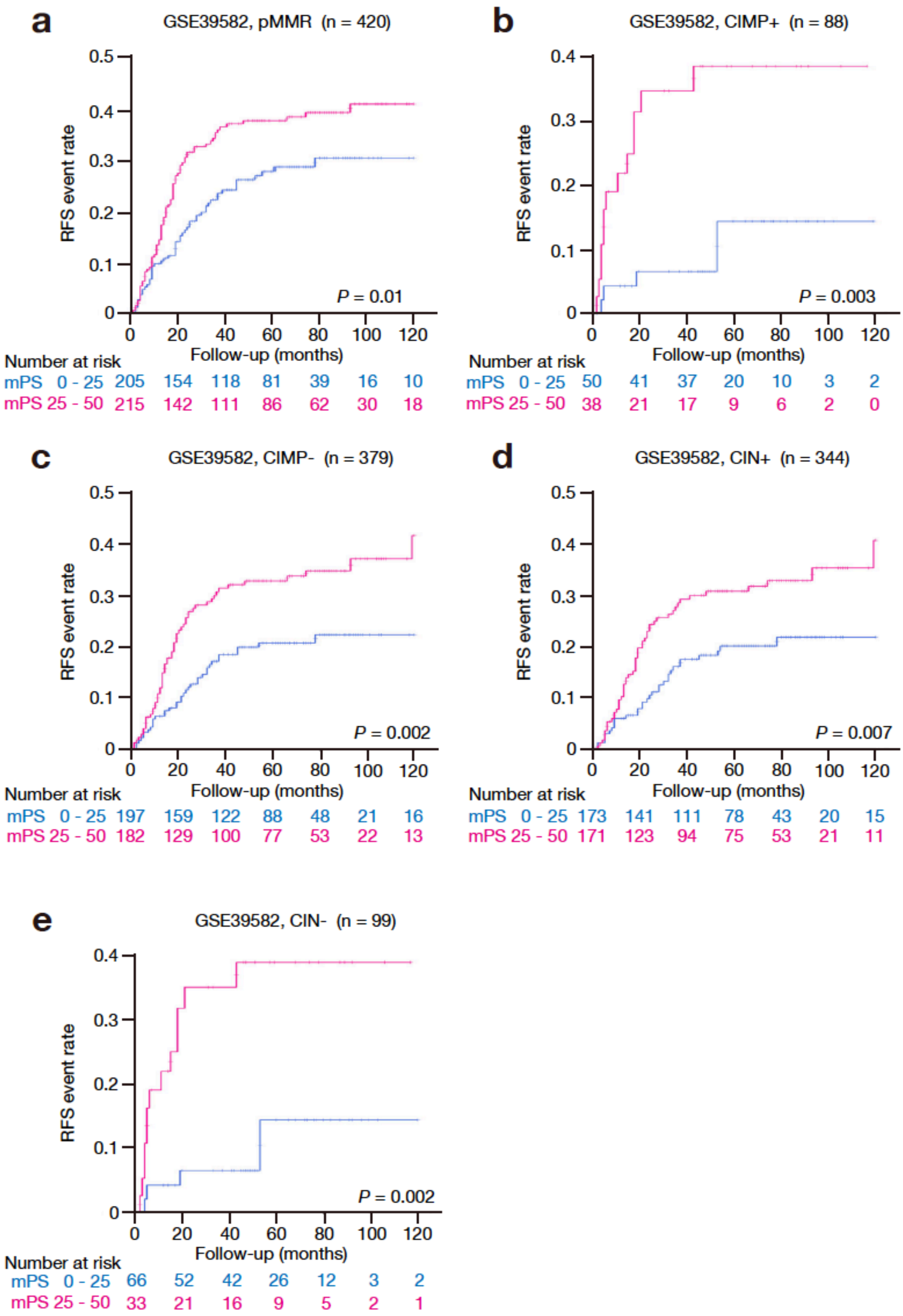

Shimizu et al., Supplementary Fig. 2

Supplementary Fig. 2 Application of mPS_colon to broad colorectal cancer subtypes. Kaplan-Meier curves of relapse-free survival (RFS) according to mPS_colon are shown for patients in the GSE39582 cohort with mismatch

repair–proficient (pMMR) tumors (**a**), CpG island methylator phenotype (CIMP)–positive (**b**) or –negative (**c**) tumors, or chromosome instability (CIN)–positive (**d**) or –negative (**e**) tumors. The log-rank *P* values are indicated. Only patients with available information are included.
